# Prognostic Scoring Systems for Burns: A Comparative Analysis of Their Predictive Accuracies for Mortality in Burn Patients

**DOI:** 10.3390/ebj7010018

**Published:** 2026-03-19

**Authors:** Susanne Rein, Jule Schmiechen, Jochen Gille, Thomas Kremer

**Affiliations:** 1 University Hospital Hamburg-Eppendorf, Martinistraße 52, 20246 Hamburg, Germany; 2 Department for Hand-. Plastic and Microsurgery, Burn Unit, Berufsgenossenschaftliches Klinikum, Bergedorfer Straße 10, 21033 Hamburg, Germany; 3 Department of Anesthesiology, Intensive Care Medicine and Pain Therapy, Klinikum St. Georg, Delitzscher Straße 141, 04129 Leipzig, Germany; 4 Department for Plastic and Hand Surgery, Burn Unit, Klinikum St. Georg, Delitzscher Straße 141, 04129 Leipzig, Germany

**Keywords:** burn, injury, morbidity, mortality, scores, survival rate

## Abstract

Introduction: Various scoring systems are applied to burn patients to assess the perioperative and mortality risks as well as comorbidities. Objective: The purpose of this study was to compare the predictive accuracies for mortality of different scoring systems: the Abbreviated Burn Severity Index (ABSI), Bogenhausen ABSI (BABSI), American Society of Anesthesiologists (ASA) classification, Charlson Comorbidity Index (CCI) and modified Frailty Index-5 (mFI-5). Materials and Methods: We retrospectively analyzed 644 burn patients treated at one burn center between September 2018 and May 2022. Results: Median scores were 5 (range: 1–16), 5 (range: 2–17.5), 2 (range: 1–5), 0 (range: 0–14) and 0 (range: 0–5) for the ABSI, BABSI, ASA, CCI and mFI-5, respectively. Significantly different median score results were observed between survivors and non-survivors: ABSI: 5 vs. 10; BABSI: 5 vs. 10.5; ASA: 2 vs. 4; CCI: 0 vs. 5; and mFI-5: 0 vs. 2 (*p* < 0.001 for all scores). Predictive accuracies were excellent for the BABSI (AUC = 0.963), ABSI (AUC = 0.952), and ASA (AUC = 0.916), whereas fair predictive accuracies were found for the CCI (AUC = 0.851) and mFI-5 (AUC = 0.760). Good calibration was observed for the BABSI, ABSI, CCI, and mFI-5, whereas calibration was poor for the ASA. Conclusion: All five scores significantly differentiate between survivors and non-survivors. However, the strongest discriminatory power and best calibration for mortality prediction were observed for the BABSI and ABSI scores. Therefore, the application of both scores is recommended in daily routine.

## 1. Introduction

Globally, burn injuries are commonly observed [1]. In Germany, 1500 extensive burn victims are treated in 22 burn centers per year [2]. Massive burn injuries lead to high morbidity and mortality requiring specialized intensive care and surgical treatment [3]. Various scoring systems are available to assess the individual perioperative and mortality risks as well as comorbidities in burn patients to estimate the likelihood of death [4]. Such models may help to guide patient care and may allow for the benchmarking of patient outcomes in different burn centers [5].

The Abbreviated Burn Severity Index (ABSI) was first described by Tobiasen and colleagues in 1982 and is routinely applied in many burn centers [6,7,8,9]. Parameters such as age, sex, total burn surface area (TBSA), presence of full-thickness burns, and inhalation trauma (IHT) are assessed. The score result and mortality risk are well correlated [7]. Multiple validations and modifications of the ABSI score have been presented [6,8,10,11,12]. One of these modifications is the Bogenhauser ABSI (BABSI), which additionally includes pre-existing health conditions, such as cardiovascular, pulmonary, renal, gastro-intestinal, and endocrinological diseases and abuse of alcohol, nicotine, and drugs [13]. Another perioperative risk assessment tool is the physical status classification system of the American Society of Anesthesiologists (ASA). This allows for a simple categorization of a patient’s physiological status. The grading system differentiates six categories: ASA-I confirms a healthy patient, ASA-II mild systemic diseases, ASA-III severe systemic diseases, ASA-IV severe systemic diseases that are a constant threat to life, ASA-V moribund patients, who do not survive without surgical treatment, and, finally, ASA-VI diagnosed brain-dead patients, who are organ donors [14,15]. Another score used to predict mortality is the Charlson Comorbidity Index (CCI). It does not focus on burn patients but is still one of the best-studied and validated scores that is most frequently applied [16,17]. The score divides 19 comorbid conditions into four severity groups. According to the severity, comorbidities are rated with 1 to 6 points. In addition, one point is added for each decade of life after the age of 50 years, resulting in a total age-adapted score of pre-existing medical conditions. The score correlates with the severity of comorbidities and subsequently with the mortality risk, and it has been used in different studies to prognostically assess mortality in patients suffering from various diseases, such as breast cancer, HIV infection and pneumonia, as well as lower-limb amputation [16]. Furthermore, the scoring system has been used to predict the length of hospitalization and the likelihood of hospital readmission [16]. The modified Frailty Index-5 (mFI-5) predicts the mortality associated with frailty. The latter is defined as an increased susceptibility of patients to illness or injury due to impaired functions of several body systems. The mFI-5 includes the following parameters: diabetes mellitus, congestive heart failure, chronic obstructive pulmonary disease, arterial hypertension requiring medication, and totally or partially dependent functional health status [18,19,20].

The high variability in the described scoring systems, each focusing on different aspects, such as burn-specific parameters, comorbidities and frailty, raises the question of the predictive value for mortality risk assessment in burn patients. The accurate prediction of the in-hospital mortality in burn patients is essential to guide clinical decision making, optimize the use of intensive care resources, and improve patient outcomes. Comparing established scoring systems may help to identify the most reliable tool to discriminate and recognize high-risk patients. Burn-specific scoring systems have been widely evaluated, and their predictive values for mortality have been compared [8,21,22]. However, comparisons with comorbidity- and frailty-based scores within the same burn patient cohort remain limited [23]. The scoring systems included in this study were selected to represent different conceptual approaches to mortality prediction, including burn-specific severity (ABSI and BABSI), comorbidities (CCI), frailty (mFI-5), and perioperative risk assessment (ASA). The aim of this study was to compare the performances of the ABSI, ASA, BABSI, CCI, and mFI-5 in the prediction of in-hospital mortality in adult burn patients. It is hypothesized that these five scores have independent predictive values for in-hospital mortality rates with different accuracies and calibrations in burned patients.

## 2. Materials and Methods

This study was approved by the local ethics committee review board (approval number EK-BR-35/22-1).

### 2.1. Study Design and Objective

We performed a retrospective cohort study at our burn center between September 2018 and May 2022. During the study period the surgical and intensive care team and treatment standards were stable. The objective of this study was to evaluate and compare the predictive accuracies of the ABSI, ASA, BABSI, CCI, and mFI-5 for in-hospital mortality in adult burn patients.

### 2.2. Patients

A cohort of 688 burned patients were recruited from our burn center. Patients that were under the age of 18 years (n = 14; 31.8%) or were admitted to the burn center more than 24 h after the injury (n = 20; 45.5%) were excluded from the analysis. Likewise, first-degree burns and polytraumatized patients were not analyzed (n = 10; 22.8%). There was no limitation for the TBSA. After exclusion, 644 patients remained for analysis. Demographic data such as age, sex, and body mass index, as well as burn-related variables, including the total body surface area, burn depth, and presence of inhalation trauma, were collected. IHT was diagnosed by bronchoscopy. In addition, mortality data and the variables required for calculating the ABSI, BABSI, ASA, CCI, and mFI-5 scores were recorded.

### 2.3. Questionnaires

The ABSI, BABSI, ASA, CCI, and mFI-5 were retrospectively calculated from the collected data and treatment charts as described previously by the authors [7,13,14,17,18]. Pseudo-anonymized data were compiled in an Excel spreadsheet database and then transferred to the statistical software SPSS (version 29.0.2.0; IBM, Armonk, NY, USA).

### 2.4. Statistics

Continuous variables, including age, TBSA, and all scoring systems (ABSI, BABSI, ASA, CCI, mFI-5), were expressed as medians with ranges due to non-normal distributions. Graphical methods and Kolmogorov–Smirnov tests were used to assess the data distribution for normality. Categorical variables, including sex, burn degree, IHT, and mortality, were presented as counts and percentages.

Bivariate analyses were performed to compare the median ABSI, ASA, BABSI, CCI, and mFI-5 scores between survivors and non-survivors using the Mann–Whitney U test. Logistic regression analyses were performed to evaluate the association between each score and the in-hospital mortality. The ASA and mFI-5 were adjusted for age, sex, TBSA, and IHT. For the age-adjusted CCI, age was not included as an additional covariate. For the ABSI and BABSI, which already incorporate age, sex, TBSA, and IHT in their calculations, these variables were not entered separately into the regression models. The predictive accuracy of the scores was determined with receiver operating characteristic (ROC) curve analysis. The Area Under the Curve (AUC) was calculated to assess the ability of each score to discriminate between survivors and non-survivors, where 0.5 indicates no discrimination and 1.0 indicates perfect discrimination. Optimal cut-off points were identified using Youden’s index. The cut-off values represent the points at which the scores best differentiate between survivors and non-survivors. The calibration of the scores was assessed with the Hosmer–Lemeshow test, which compared predicted mortality probabilities to actual outcomes. A higher *p*-value from the Hosmer–Lemeshow test indicates better calibration, suggesting that the predicted probabilities are closely aligned with the actual outcomes. Descriptive and statistical tests were conducted using SPSS version 29.0.2.0 (20) (IBM, Chicago, IL, USA). Statistical significance was set at *p* < 0.05.

## 3. Results

### 3.1. Demographic Data

Four hundred forty-one (68.5%) patients were male, and 203 (31.5%) were female. The median age of the patients at the time of their injury was 44 years (range: 18–93 years). A total of 25 (3.9%) out of 644 patients died during hospitalization. The median TBSA was 3.5% (range: 0.01–100%), with the burn grades distributed as followed: 35 patients (5.4%) with superficial partial thickness (grade 2a) burns; 277 patients (43.0%) with deep partial thickness (grade 2b) burns; 332 patients (51.5%) with full-thickness (grade 3) burns. Inhalation injury was diagnosed in 34 patients (5.3%).

### 3.2. Bivariate Analysis

**Figure 1:**
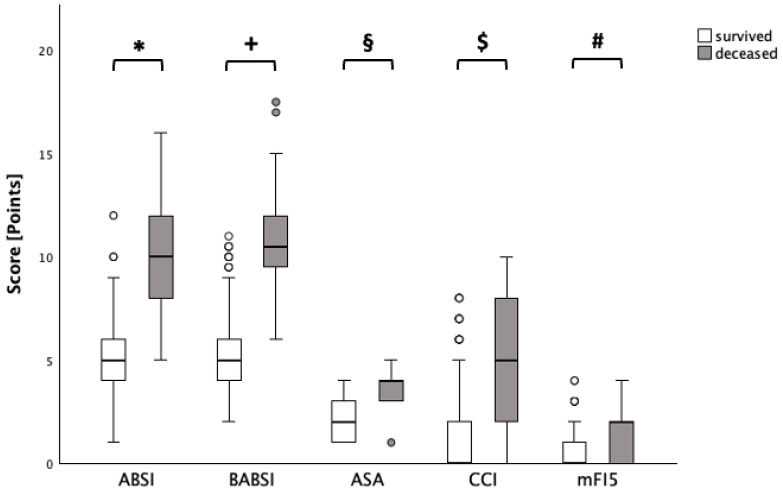
Mortality outcomes. The graph shows the results of the bivariate analysis between deceased and survived patients for the different scoring systems as box plots. The ABSI (*), BABSI (+), ASA (§), CCI ($), and mFI-5 (#) significantly differ between deceased and survived burn patients (*p* < 0.001). o—data points lying beyond 1.5 times the interquartile range (IQR) from the first or third quartile.

The median and the minimum and maximum were obtained from the different scores: ABSI: 5 (range: 1–16); ASA: 2 (range: 1–5); BABSI: 5 (range: 2–17.5); CCI: 0 (range: 0–10); and mFI-5: 0 (range: 0–5). Mann–Whitney U tests showed statistically significant differences in the ABSIs between survivors (median = 5) and non-survivors (mean = 10; *p* < 0.001, r = 0.31), as well as in the ASA classifications (survived: median = 2; deceased: median = 4; *p* < 0.001, r = 0.30), BABSIs (survived: median = 5; deceased: median = 10.5; *p* < 0.001, r = 0.30), CCIs (survived: median = 0; deceased: median = 5; *p* < 0.001, r = 0.24) and mFI-5 scores (survived: median = 0; deceased: median = 2; *p* < 0.001, r = 0.18) (Figure 1).

### 3.3. Logistic Regression Analyses

**Figure 2:**
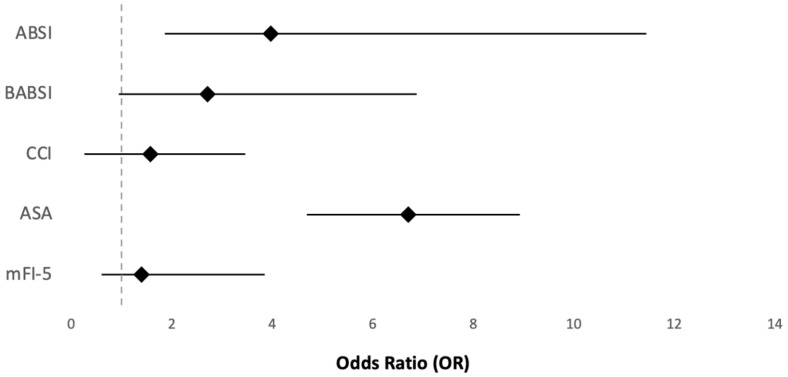
Forest plot of odds for mortality. Diamonds represent estimated points of odds ratios (ORs) for ABSI, BABSI, CCI, ASA, and mFI-5. Horizontal lines indicate 95% confidence intervals (CIs). Dashed vertical line at OR = 1.0 represents line of no effect. ASA has highest odds of mortality, followed by ABSI and BABSI.

Logistic regression analyses adjusted for age, sex, TBSA, burn degree, and IHT showed that the ASA score was statistically significantly associated with mortality (*p* = 0.02). For each category increase in the ASA score, the odds of mortality increased by a factor of 6.7, accounting for the other covariates (Figure 2). The logistic regression analysis also showed statistically significant associations between the ABSI, BABSI and CCI and mortality (*p* < 0.001). For each unit increase in the ABSI, the odds of mortality increased by 4.0. For the BABSI, the odds increased by 2.7, and for the CCI, they increased by 1.6. The logistic regression analysis did not show a statistically significant association between the mFI-5 and mortality (Figure 2).

### 3.4. ROC Curve Analysis and Hosmer–Lemeshow Tests

**Figure 3:**
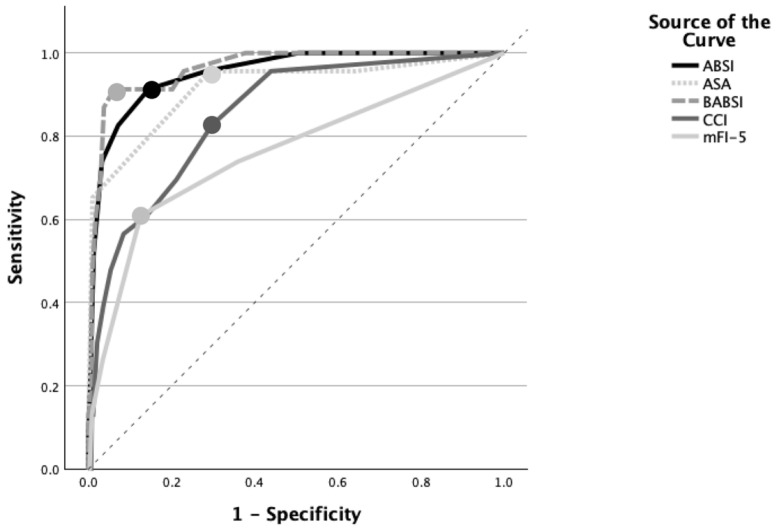
Receiver operating characteristic curves of different scoring systems to predict mortality. ROC curves for prediction of mortality of ABSI, ASA, BABSI, CCI, and mFI-5 are presented. Dashed diagonal line indicates reference line (AUC = 0.5), indicating no discrimination. Dots along each curve symbolize optimal cut-off points according to Youden index.

The ROC curve analysis showed that the ABSI (AUC = 0.952, SE 0.019, *p* < 0.001), BABSI (AUC = 0.963, SE 0.016, *p* < 0.001), and ASA (AUC = 0.916, SE = 0.037, *p* < 0.001) exhibited excellent predictive accuracies for mortality, while the CCI (AUC = 0.851, SE = 0.038, *p* = 0.002) and mFI-5 (AUC = 0.760, SE = 0.060 0, *p* < 0.001) had fair accuracies (Figure 3). All five scores were statistically significantly associated with mortality. The BABSI had an optimal cut-off value of 8.25 (Youden’s index: 0.84), the ABSI a score of 6.5 (Youden’s index: 0.77), the ASA a score of 2.5 (Youden’s index: 0.67), the CCI a score of 1.5 (Youden’s index: 0.530), and the mFI-5 a score of 1.5 (Youden’s index: 0.483), indicating that these values best differentiate between survivors and non-survivors.

The Hosmer–Lemeshow test was used to assess the calibrations of the different scores. Lower Chi-square values and higher *p*-values indicate better calibration, with *p* > 0.05 suggesting no significant difference between predicted and observed outcomes. The results indicate that the BABSI (Chi-square = 0.900, *p* = 0.996) and ABSI (Chi-square = 1.195, *p* = 0.945) exhibit excellent calibrations, suggesting that the predictions closely match the observed data. This reflects a very good agreement of the predicted and actual mortality rates. The mFI-5 (Chi-square = 1.962, *p* = 0.161) and CCI (Chi-square = 4.020, *p* = 0.259) also demonstrated good calibrations, indicating reliable predictive accuracies. In contrast, the ASA score model shows significant deviation (Chi-square = 81.133, *p* < 0.001), pointing to poor calibration and suggesting that its predictions do not align well with actual outcomes.

## 4. Discussion

### 4.1. Scores

This study demonstrates that all scores significantly discriminate between survivors and non-survivors. Furthermore, the highest odds of mortality were found for the ASA, followed by the ABSI and BABSI. The mFI-5 had no significant odd for mortality. Finally, the BABSI and ABSI had the strongest discriminatory power and the best calibration to predict mortality in adult burn patients.

A meta-analysis, including 16 studies of the ABSI, reported a mean AUC of 0.89, whereas the AUC for mortality prediction in the present study was 0.95 [24]. The ABSI has been criticized in the literature because pre-existing medical conditions or drug abuses in burn patients are not considered and mortality in older and/or critical care patients may be underestimated [25,26]. Therefore, the BABSI was developed, which includes patients’ comorbidities. An AUC of 0.96 was observed, which is comparable to the AUC of the ABSI (0.95).

These results are comparable to those of Hörbrand et al., who developed the BABSI and reported AUCs of 0.89 for the ABSI and 0.90 for the BABSI [13]. In contrast, scores focusing on comorbidities, such as the CCI and mFI-5, showed poorer mortality predictions (AUCs of 0.85 and 0.76, respectively). Neither of the scores include burn-specific parameters, such as the TBSA or degree of burn. Another study introduced a modified ABSI, excluding sex and using a modified point scale for age. An AUC of 0.96 was observed [6]. Recently, the odds of death were reported to increase by a factor of 2.059 with each additional ABSI point in a retrospective study on 1193 burn patients [27]. The effect observed in the present study was even higher with a factor of 4.0. These differences could be explained by the fact that Christ et al. used an univariate analysis [27], whereas a multivariate analysis was performed in the present study, which was adjusted for age, sex, TBSA, burn degree and IHT.

The Hosmer–Lemeshow test revealed a cut-off point of 6.5 for the ABSI in the present study. Likewise, an Indonesian study reported a cut-off point of 6.79 [28]. However, weak calibration of the ABSI was reported in a retrospective study of 9625 burn patients in China. Here, the ABSI was observed to overestimate mortality in a 10-year study period [12]. The discrepancy in the calibrations of the ABSI between our study (Chi-square = 1.195, *p* = 0.945) and the study of Zhou et al. (Chi-square = 1198.97, *p* < 0.001) might be explained by the sensitivity of the Hosmer–Lemeshow test to large sample sizes. Here, even minimal differences between observed and predicted outcomes lead to statistically significant results without necessarily indicating poor model calibration [12,29]. In summary, the original ABSI still has sufficient prediction value for mortality in burn patients based on our results as well as the literature [28,30,31].

An AUC of 0.71 was observed for the predictive accuracy of the ASA in a study of 183 adult burn patients, whereas a poorer value of 0.91 was observed in the present study [28]. The smaller sample of 183 patients reduces the statistical power, and stricter inclusion criteria, such as patients with TBSAs ≥ 10% undergoing surgery, may have led to bias. A retrospective multicenter study of 3042 trauma patients reported an AUC of 0.886 for the ASA, which is comparable to the AUC of 0.91 in the present study [5]. However, the study states an odds ratio of 4.53 for the ASA, whereas a higher odds ratio of mortality of 6.7 was observed in the present study [5]. The reason for these different results may be the different patient populations, since burn patients may have a higher mortality risk with increased ASA categories compared to trauma patients.

Furthermore, the revised Baux score and updated CCI were independently associated with mortality in 90 burn ICU patients with an AUC of 0.92 [32], which is different from the present results with an AUC of 0.68 for the CCI. This may be explained by the fact that the revised Baux score and updated CCI were combined for the ROC curve analysis by Heng and colleagues [23], whereas our ROC analysis was calculated for each score separately.

Results for the mortality prediction of the CCI remain controversial. The mortality of 7640 burn victims was assessed by Heng et al., and the OR was observed to increase by 1.59 for each point increase in the CCI [23]. This was similarly observed in the present study, which showed an OR of 1.6. In 2011, a large study on 55,929 patients of various specialties was performed to evaluate the in-hospital mortality according to the CCI in six countries [33]. A range from 0.727 to 0.882 of the AUC was observed for predictions of the in-hospital, 30-day and 1-year mortality [34]. In contrast, our study showed a lower AUC of 0.68. This indicates that prediction of mortality using the CCI is less effective in burn patients when compared to other specialties. An additional study evaluated the factors influencing in-hospital mortality in burn patients. Based on the CCI results, the study showed that age alone was associated with increased in-hospital mortality rates, whereas underlying comorbidities showed no significant impact on mortality [35]. A recent study performed a multivariate logistic regression analysis on 617 burn patients, showing that mFI-5 scores of ≥2 do not represent an independent risk factor for in-hospital mortality. The authors concluded that the mFI-5 is not a reliable predictor for in-hospital mortality [20], which was likewise observed in the present study. However, to the best of our knowledge, no studies are available up to now that have calculated the AUC of the mFI-5 for its predictive power of mortality in burn patients. Sen et al. evaluated 347 burn patients in 2023 [36]. Here, the mFI-5 was observed to be an independent predictor of mortality. With an increase in each mFI-5 point, the odds for mortality were shown to increase by 1.85, which is in contrast to the results of the present study [36]. A study conducted on 4662 patients who underwent spinal tumor surgery showed an AUC of 0.74 for the mFI-5, which is a very similar predictive accuracy for mortality to the 0.76 in this study [37].

These findings show that burn-specific scores provide the most reliable mortality prediction, whereas comorbidity- and frailty-based scores may offer additional context on patient vulnerability but are less predictive of in-hospital mortality.

### 4.2. Study Limitations and Further Directions

A retrospective study at a single burn center was conducted, limiting the ability to generalize the results based on ecoregional treatment algorithms and access to care. Furthermore, the documentation quality depended on information availability in medical records. Comorbidities may have been missed, misclassified, or underestimated. Furthermore, only in-hospital mortality was analyzed in the present study. Additionally, prognostic scores fail to predict long-term health-related issues and patient-reported outcomes, such as quality of life. Additionally, the relatively low number of in-hospital mortality events represents an important methodological limitation. Multivariable logistic regression models based on a limited number of outcome events may be prone to overfitting and unstable effect estimates. External validation in larger, independent cohorts is warranted to confirm the robustness and generalizability of the observed associations. Furthermore, calibration was assessed using the Hosmer–Lemeshow test only. Future studies should incorporate additional graphical and quantitative calibration measures to enable a more comprehensive evaluation of model performance.

Furthermore, only a limited number of burn outcome predictive models were selected. However, additional scores exist, such as the Baux score [38], burn mortality prediction score (BUMP) [1] and Belgian Outcome of Burn Injury (BOBI) [39], and were not included in this study. In addition to the well-established scores, machine learning-based approaches have been introduced in burn mortality prediction. For example, the Bochum Burn Survival Score [40] applies modern machine learning algorithms to predict survival in burn patients. It is important to note that mortality prediction tools are usually inaugurated in highly developed countries with highly technical resources, particular in cases of specialized burn care, and they can provide an accurate prediction of mortality in different patient groups [12].

Future research is required to validate the burn-specific, comorbidity-, and frailty-based scores across diverse healthcare systems and countries with varying resources to assess their generalizability. The potential to combine comorbidity-and frailty-based scores with burn-specific parameters, such as the TBSA, the burn depth, and inhalation injury, should also be explored to improve individualized risk assessment. In addition, studies should examine the predictive value of these scores beyond in-hospital mortality by assessing long-term outcomes such as survival, functional recovery, and health-related quality of life. Furthermore, the results of the different scores should be analyzed in the context of the standardized mortality rate (SMR) [41] or variable life-adjusted display (VLAD) [42], which allows for the reporting of the quality of care in burn centers. Both scores allow for the observation of smaller and shorter-term variations in the difference between the observed and predicted mortality [41]. Finally, the integration of prognostic scores into clinical protocols and decision-making pathways should be evaluated to understand their potential impact on patient management and the quality of burn care.

## 5. Conclusions

Although all five scores significantly differentiate between survivors and non-survivors, the results show that both the BABSI and ABSI have the strongest discriminatory power and best calibration to predict mortality in adult burn patients. Therefore, the application of both established scores is recommended in daily routine. The mFI-5 and CCI reflect the severity of comorbidities but show weak predictions for mortality in adult burn patients. The ASA has the highest odds of mortality for each higher category in burn patients.

## Data Availability

The data presented in this study are available upon request from the corresponding author due to patient confidentiality and the potential risk of re-identification despite anonymization.

## Abbreviations

The following abbreviations are used in this manuscript:

ABSI	Abbreviated burn severity index
ASA	American Society of Anesthesiologists physical status classification
AUC	Area under the curve
BABSI	Bogenhausen abbreviated burn severity index
BOBI	Belgian outcome of burn injury
BUMP	Burn mortality prediction score
CCI	Charlson comorbidity index
CI	Confidence interval
ICU	Intensive care unit
IHT	Inhalation trauma
IQR	Interquartile range
mFI-5	Modified frailty index-5
OR	Odds ratio
ROC	Receiver operating characteristic
SE	Standard error
SMR	Standardized mortality rate
TBSA	Total burn surface area
VLAD	Variable life-adjusted display
